# Iterative sure independent ranking and screening for drug response prediction

**DOI:** 10.1186/s12911-020-01240-9

**Published:** 2020-09-22

**Authors:** Biao An, Qianwen Zhang, Yun Fang, Ming Chen, Yufang Qin

**Affiliations:** 1 Department of Mathematics, Shanghai Normal University, Shanghai, China; 2 College of Information Technology, Shanghai Ocean University, Shanghai, China; 3 Key Laboratory of Fisheries Information Ministry of Agriculture, Shanghai, China

**Keywords:** SIRS, Drug response, ISIRS, CCLE

## Abstract

**Background:**

Prediction of drug response based on multi-omics data is a crucial task in the research of personalized cancer therapy.

**Results:**

We proposed an iterative sure independent ranking and screening (ISIRS) scheme to select drug response-associated features and applied it to the Cancer Cell Line Encyclopedia (CCLE) dataset. For each drug in CCLE, we incorporated multi-omics data including copy number alterations, mutation and gene expression and selected up to 50 features using ISIRS. Then a linear regression model based on the selected features was exploited to predict the drug response. Cross validation test shows that our prediction accuracies are higher than existing methods for most drugs.

**Conclusions:**

Our study indicates that the features selected by the marginal utility measure, which measures the conditional probability of drug responses given the feature, are helpful for drug response prediction.

## Background

A major goal in cancer research is to select an efficacious drug or drug combinations for each individual patient based on their genomic and transcriptomic profiles [1]. To get a much more comprehensive understanding of the potential genetic makeup of a patient, researchers have tried multi-omics data including protein concentration, gene expression and genetic mutations. However, the methodology of translating the genetic measurements to predictive models for assisting therapeutic decisions is still a challenge.

Researchers have tried many methods to find biomarkers and predict drug sensitivity. These methods are mainly based on gene expression measurements. Staunton et al. proposed a weighted voting classification strategy to classify each cell line as sensitive or resistant for each drug based on the NCI-60 gene expression data [2]. Riddick et al. developed a novel multistep regression model for drug response using Random Forest [3]. However, the biomarker of a certain drug for different cancer types may be different because of the heterogeneity of different cancers, so it is more realistic to focus on some specific type of cancers. Lee et al. developed a genetic algorithm termed as “coexpression extrapolation”, which can accurately predict drug sensitivity of bladder cancer cell lines and clinical responses of breast cancer patients treated by commonly used chemotherapeutic drugs [4]. Holleman et al. used 14,500 probe sets to identify differentially expressed genes in drug-sensitive and drug-resistant acute lymphoblastic leukemia (ALL) [5]. Besides gene expression, some researchers paid attention to the related factors including epigenetic modifications, chemical description of the drugs and so on. Shen et al. used bisulfite PCR to assess DNA methylation and employed the methylation markers to predict drug sensitivity [6]. Chen et al. proposed a graph matching with multiple network constraints model to accurately identify gene-drug modules [7]. Wang et al. used elastic net regression to report the relationship between the lncRNA pharmacogenomic landscape by integrating multi-dimensional genomic data and drug response data [8]. Additionally, Menden et al. [9] developed a machine learning model to predict the response of cancer cell lines to drug treatment based on both the genomic features of cell lines and chemical properties of the considered drugs. In spite of the success in finding some drug biomarkers, these kinds of approaches still suffer from the typical problem of “high-dimension but low sample size” problem in statistical learning, i.e., compared with the large number of expression genes and chemical compounds (p), the number of samples (n) is very limited.

In a recent study, researchers from the Broad Institute of Harvard and MIT generated a large scale genomic data for cancer cell lines (termed as the Cancer Cell Line Encyclopedia, CCLE). Coupling with pharmacological profiles for 24 anticancer drugs across 479 cell lines, this dataset could allow identification of genetic, lineage, and gene-expression-based predictors of drug sensitivity [10]. In this paper, the authors first screened all genomic features by their marginal correlation with drug response, and then predicted drug sensitivity by elastic net regression. However, the interaction between genomic features also influences drug sensitivities, so the importance of features may change after adding other features into the model. In order to incorporate the important features with weak marginal correlation and remove the marginally strong but jointly unimportant features, Fang et al. applied an iterative sure independence screening (ISIS) to CCLE dataset and improved the accuracy for drug sensitivity prediction [11]. However, the feature screening based on Pearson correlation coefficient (PCC) is sensitive to outliers and needs the assumption of elliptically symmetric distribution in theory [12]. Considering the existence of outliers and asymmetric distribution for most drug sensitivity data in CCLE, we exploited the sure independent ranking and screening (SIRS) [13] that measures the conditional distribution of drug response given genomic features. Note that Zhu et al. [13] proposed a model-free feature screening approach SIRS to select important features. The SIRS method used the residual of remaining features to do the iterations. To predict the drug response through using the identified important features, we exploit the linearity assumption in modeling drug response and use the residual of response instead of the residual of remaining features to do the iterations.

In this paper, we propose the iterative SIRS (ISIRS) to predict the drug response and apply it to the CCLE dataset. By using the iterative procedure of ISIRS, strong features with marginally weak utility measures can have chance to be recruited, and the weak features with marginally strong measures can be removed. The cross-validation tests showed that the prediction accuracies of our method outperformed ISIS, STF and SIRS for most drugs in CCLE dataset. Compared with ISIS, the feature importance by PCC showed that ISIRS is robust to outliers and releases the assumption of symmetric distribution. Additionally, we also detected some new drug response related genomic features.

## Methods

### Datasets

The drug response and cancer genomic data used in this present paper are available from the Cancer Cell Line Encyclopedia (CCLE). This dataset contains copy number alteration, gene expression and mutation status for 947 human cancer cell lines, as well as 8-point dose-response curves for 24 chemical compounds across 479 cell lines. We used the area under dose-response curves (termed as Activity area in [10]) instead of EC50 and IC50 to measure the sensitivity of drug for a given cell line. It has been extensively exploited because of its efficacy and potency of characterize a drug [14].

### Screening procedure

For each cell line, expression profile of 20,069 genes, mutation status of 1654 genes and copy number status of 16,045 genes are integrated as the primary feature vector. The dimension of primary feature vector is too high compared with the sample size. In this paper, the sure independent ranking and screening [13] was introduced to marginally select drug response-related features.

Let ***Y*** be the drug response value and ***x*** = (*X*_1_, …, *X*_*p*_)^T^ be the vector consisting of all candidate features. Without loss of generality, we assume that *E*(*X*_*k*_) = 0 and *var*(*X*_*k*_) = 1 for *k* = 1, …, *p* after scaling. As suggested in reference [13], we denote the conditional distribution function of ***Y*** given ***x*** by *E* (*y* **| x**) = *P* (***Y*** *< y* ***|x***) and define ***Ω***(*y*) = *E*{***x****E*(*y*| ***x***)}. Let *Ω*_*k*_(*y*) be the *k-*th element of ***Ω***(*y*) and *ω*_*k*_ is defined as

$$ {\omega}_k=E\left\{{\varOmega}_k^2\ \left(\boldsymbol{Y}\right)\right\},k=1,\dots, p. $$

In the present paper, we take ω_k_ as the marginal utility measure. The predictor X_k_ is called active predictor if E (y | x) functionally depends on X_k_, and the one which E (y | x) does not relate with is referred as inactive predictor [13]. Due to the consistency in ranking, the marginal utility measure *ω*_*k*_ can always rank an active predictor above an inactive predictor. This guarantees that we can select the active predictors and exclude all inactive predictors [13]. Thus we used *ω*_*k*_ for feature screening. For real data, the sample counterpart of *ω*_*k*_ is obtained as follows.

Let {(***x***_*i*_, ***Y***_*i*_), *i* = 1, …, *n*} be a random sample of {***x***, ***Y***}. First, we normalized the sample predictors such that ${n}^{-1}{\sum}_{i=1}^n{X}_{ik}=0$ and ${n}^{-1}{\sum}_{i=1}^n{X_{ik}}^2=1$ for *k* = 1, …, *p*. Then the sample estimator for *ω*_*k*_ is.

$$ {\hat{\omega}}_k=\frac{1}{n}\ {\sum}_{j=1}^n{\left\{\frac{1}{n}\kern0.5em {\sum}_{i=1}^n{X}_{ik}\boldsymbol{I}\ \left({\boldsymbol{Y}}_i<{\boldsymbol{Y}}_j\right)\right\}}^2, $$*k* = 1, …, *p,*

where *X*_*ik*_ is the *k*th component of ***x***_*i*_. According to the descending order of *ω*_*k*_, all the candidate predictors (features) can be ranked and the top ones are recruited.

### Feature selection through iterative sure independent ranking and screening

All feature screening methods based on marginal utility measure suffer from an inherent drawback that they may miss the predictors that are marginally insignificant but jointly related with the response. Based on this observation, we further propose the scheme of iterative sure independent ranking and screening (ISIRS) as follows.

First, we rank all features by sorting ${\hat{\omega}}_k$ as above in descending order and select the top *K*_1_ features as A_1_. Then we carry out the lasso regression based on a linear model for variable selection and get a subset *M*_1_ of *A*_1_*.* That is, we minimize the objective function

$$ L\left({\beta}_0,{\beta}_{k,k\in {A}_1}\right)={\sum}_{i=1}^n{\left({Y}_i-{\beta}_0-{\sum}_{k\in {A}_1}{\beta}_k{X}_{ik}\right)}^2+\lambda {\sum}_{k\in {A}_1}\mid {\beta}_k\mid, $$

where *X*_*ik*_ is the *k*-th component of the feature vector ***x***_*i*_*, Y*_*i*_ is the *i*-th observation of drug response, *β*_0_ and *β*_*k*_ are lasso estimators, *n* is the sample size and λ is the penalty tuning parameter. Lasso regression gives shrinkage estimates and some *β*_*k*_ can be estimated exactly as zero. The features with nonzero coefficients will be retained with the indices set denoted by *M*_1_. We use the notation ∣*M*_1_∣ to mean the numbers of features in *M*_1_. Consequently, we fit the drug response over the features in *M*_1_ by a linear regression model and obtain the residuals. Then we take the residuals as a new response and employ SIRS to select the indices set *A*_2_ from the remaining features with the indices {1, 2, …, *p*}\*M*_1_. In the next step, for the features in the union of *A*_2_ and *M*_1_, we use the lasso regression again and get a subset of features, denoted by *M*_2_. Assume that we aim to select *d* features, the process of feature screening and selection is repeated until ∣*M*_*s*_ ∣  = *d* or |*M*_*s*_| =  ∣ *M*_*s* − 1_∣. To make sure this procedure not stop at the first iteration, we set *K*_1_ *=* $\left[\frac{2d}{3}\right]$ as suggested in [9]. Besides, the consistency in both variable selection and parameter estimation cannot be achieved by lasso at the same time. So similar to Fang et al. [11], we fit a linear regression model based on the selected features by ISIRS and predict drug response by the estimates of ordinary least squares (OLS).

### Cross-validation and evaluation

In statistical prediction, the cross-validation method is often adopted to test the effectiveness of a predictor [15]. In this paper, we performed 10-fold cross-validation to validate our algorithm. In detail, in each fold, all cell lines treated by one drug were divided into ten roughly equal groups, one of which was processed as the test dataset and the rest nine groups were as the training set to train the model. The average performance across all ten folds was chosen as the final predictive value of drug response.

The Pearson correlation coefficient (PCC) between the average of predicted values and the observed response was used to evaluate the predicting performance, which has been widely used in the literatures [16]*.* Besides the criterion of PCC, we also calculated the mean squared errors (MSE) of the averaged predicted values from the 10-fold cross validation to assess the predicting performance of ISIRS.

### T-test for the significance of regression coefficients

As we know, all features explain the response (drug sensitivity) collectively in the multiple regression models and the explanatory effect is not just the simple summation of the marginal explanatory effects. It is possible that some features may have weak marginal importance but jointly related to the response.

In order to examine the importance of these marginally weak features, the t-test approach was applied to test the significance of the corresponding regression coefficient. If the coefficient is significantly different from zero, it means that the feature is important jointly with other features. Therefore, the testing problem can be described as follows:

$$ {H}_0:{\beta}_j=\kern0.5em 0;\kern0.75em {H}_1:\kern0.5em {\beta}_j\ne 0. $$

Combining the regression model, the above testing problem is essentially equivalent to the testing model,

$$ {H}_0:{Y}_i={\beta}_0+{\beta}_1{X}_{i1}+\dots +{\beta}_{j-1}{X}_{ij-1}+{\beta}_{j+1}{X}_{ij+1}+{\beta}_d{X}_{id}; $$

$$ {H}_1:{Y}_i={\beta}_0+{\beta}_1{X}_{i1}+\dots +{\beta}_d{X}_{id}. $$

When we reject *H*_0_, it means that the model in *H*_1_ with *X*_*ij*_ can explain the response *Y*_*i*_ better than the model in *H*_0_ without *X*_*ij*_, and the existence of the feature *X*_*ij*_ is significant. In this paper, the significance level was set to be 0.01.

## Results and discussion

### Determination of the number of selected features

**Fig. 1:**
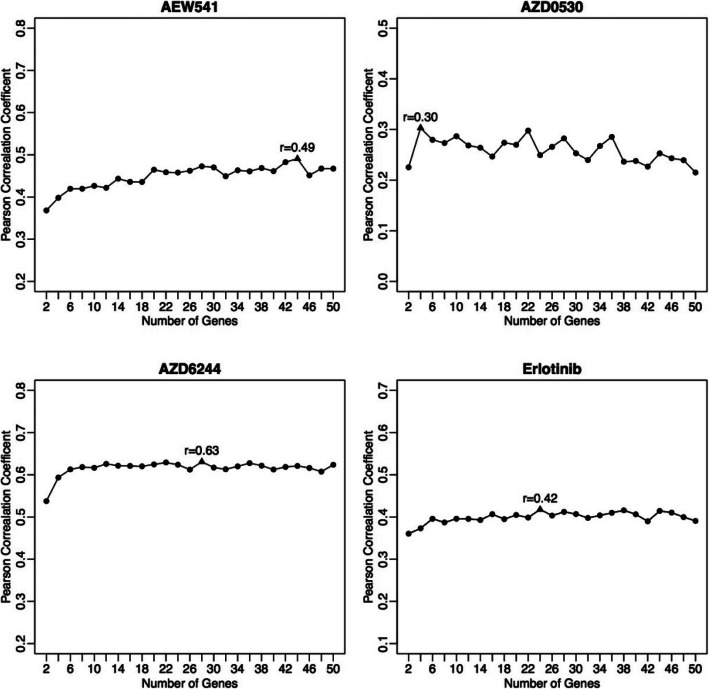
The PCCs between true and predicted drug sensitivities (DS) with different numbers of features

To determine the number of selected features *d*, we explored the predictive performance for each drug with different top features selected by ISIRS. Pearson correlation coefficient between predicted and true response values does not show significant improvement when the selected features are more than 50. This could be that the increased number of features also increases the noise. Therefore we consider the selected features less than 50 for each drug. The PCCs based on 10-fold cross validation for four example drugs are shown in Fig. 1 and the results of the rest drugs are shown in Fig. S1.

Now we set the evenly spaced grids {2, 4, 6, …, 50} for *d* and performed 10 iterations of the 10-fold cross-validation using the ISIRS scheme. The grid point corresponding to the largest PCC between the observed drug responses and predicted values via the 10-fold cross validation was taken as the optimal choice for *d.* For each drug, the final selected features are shown in Table S1.

### Analysis of selected features in ISIRS model applying in CCLE dataset

As shown in Table S1, many selected features have significant overlap with those by elastic net regression. It is also similar to elastic net regression that most selected features are gene expression data rather than mutation and copy number alteration status, which is expected since expression profile constitutes the majority of original feature source. Most of the selected features are widely accepted indicators for drug response. For example, the selected mutation features for AZD6244 and PD.0325901 include BRAF and NRAS, which are known to be the predictor of sensitivity to MEK inhibitors. Mutation of BRAF is also ranked as the top feature for PLX4720 (BRAF inhibitor). SLFN11 expression correlates with the antiproliferative activity of topoisomerase I (Top1) inhibitors in the NCI-60 [17]. As we all know, Irinotecan is DNA Topoisomerase I Inhibitor. These powerful features are also successfully selected as predictors of drug response by elastic net regression ENR [8].

The target of Paclitaxel is beta-tubulin and the mechanism of action is Microtubule-Stabilizing Agents. The gene ABCB1 was selected as a strong feature by ISIRS. The mechanisms of resistance to this class of compounds include overexpression of the drug efflux pump protein ABCB1, microtubule cytoskeletal changes, and over expression of specific β-tubulin isotype and microtubule-associated proteins. The microtubule-stabilizing agents, such as epothilones, have demonstrated similar activity in ABCB1-overexpressing cells [18]. But ABCB1 was not selected as strong features by ISIS and ENR. Nilotinib is a selective BCR-ABL tyrosine kinase inhibitor. BCR-ABL1 is positive in adult acute lymphoblastic leukemia (ALL). We selected gene IKZF1 as an active feature. It is reported that IKZF1 deletions are likely to be a genomic alteration that significantly affects the prognosis of Ph-positive ALL in adults [19]. AZD0530 is a potent Src family kinase (SFK)/Ab1 dual-kinase inhibitor. It is reported that RSF1 is an amplified gene in the highly aggressive ovarian serous carcinoma. The increased RSF1 expression and thus excessive RSF activity can induce chromosomal instability likely through DDR [20]. Gene RSF1 was also selected in our method for AZD0530. PHA.665752 is a c-MET Inhibitor. For PHA.665752, ISIRS selects mutation of RHOA as features, which could regulate the coendocytosis of cadherin and c-Met [21]. We selected mutation feature AURKC as an active feature for Panobinostat, which has been used in combination with other chemotherapy for children with relapsed AML. The AURKs are serine kinases that are involved mainly in checkpoint regulation in the cell cycle. And three mammalian AURKs have been identified: AURKA, AURKB, and AURKC [22]. Some selective inhibitors of AUIKA and AUIKB have been used in AML treatment. All these features have a common characteristic that their *ω*_*k*_ rankings are very low, but are significant according to the regression model. So we can conclude that ISIRS could detect some weak features that jointly correlate with drug response.

**Fig. 2:**
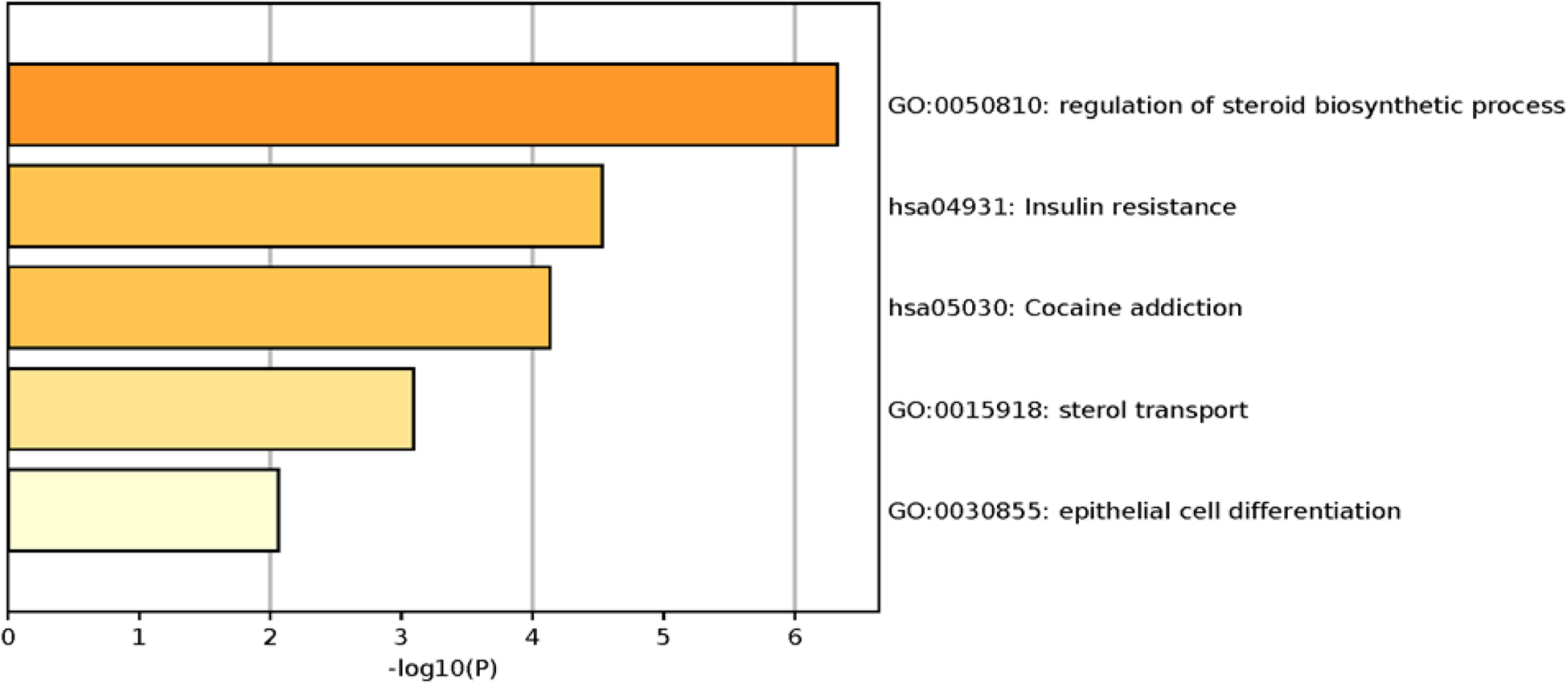
The functional enrichment analysis of the selected features for the drug Paclitaxel

To verify the relationship between drugs and the selected genes, we conducted functional enrichment analysis of the selected genes using online metascape tool [23] by taking X17.AAG as an example. The results are shown in Fig. 2. For X17.AAG, the selected 43 genes by ISIRS are significantly enriched in six function terms. The most significant GO term is regulation of steroid biosynth, which contained the genes LBH and NADH. As is reported, the steroid sulfatases convert the local inactive estrogens to their active forms, thus support the breast cancer cells growth [24]. LBH is considered an oncogene directly regulated by the Wnt/β-catenin pathway, and the overexpression of LBH leads to a more aggressive basal differentiation of breast cancer [25]. The gene NADH is known to halt the progression of breast cancer cells. This is because that NADH supplies cellular ATP and then cancer cells cannot grow in an ATP rich environment [26]. And the efficacy of the inhibitor X17.AAG was tested in breast cancer cell lines and X17.AAG was shown to inhibit the growth of breast cancer cells in vitro study [27].

### Comparison with existing methods

**Fig. 3:**
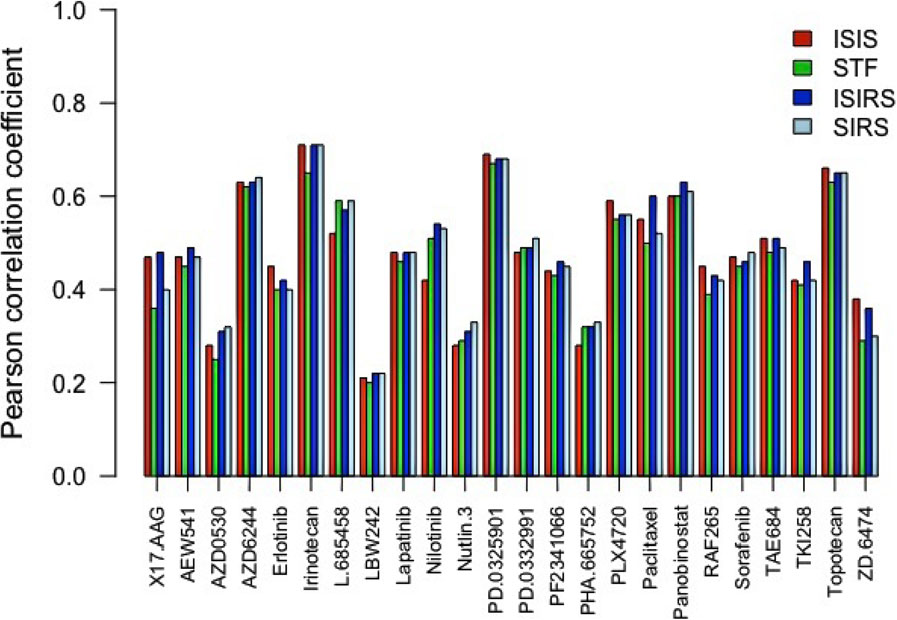
Comparison of ISIRS with ISIS, STF and SIRS in drug sensitivity prediction. Pearson correlation coefficients between predicted and true drug sensitivities by ISIS (red), STF (green), ISIRS (blue), SIRS (light blue)

To evaluate the performance of ISIRS model, we made comparisons with iterative sure independence screening (ISIS), sure independence screening (SIRS) and simple top features (STF), by choosing the Pearson correlation coefficients as the comparative measure. The Pearson correlation coefficients between true and predicted drug responses by ISIRS, ISIS, SIRS, and STF are reported in Table S2 and showed by a bar chart in Fig. 3. We could conclude that our prediction was slightly better than STF and almost the same as ISIS and SIRS. For instance, the mean increase in Pearson correlation coefficients of ISIRS was closer to 0.03 compared with STF. When comparing with ISIS and SIRS, the overall increases are about 0.014 and 0.011. Explicitly, the PCC of L.685458 has increased from 0.52 to 0.57, the increase is 0.12 for Nilotinib (from 0.42 to 0.54), the increase is 0.05 for Paclitaxel from 0.55 to 0.6 and the increase is 0.04 for TKI258 from 0.42 to 0.46 when compared with ISIS. The PCC of 17.AAG has increased from 0.40 to 0.48, and the increase is 0.08 for Paclitaxel and 0.06 for ZD.6474 when it is compared with SIRS. In addition, the predicted correlation by ISIRS are higher than those by STF, with the paired Wilcox-test (*p*-value = 7.09e-05). Also, ISIRS gives higher predicted correlations than ISIS, with the paired Wilcox-test (*p*-value = 0.02074), and the performance of STF and ISIS is comparable as expected with p-value = 0.02151 by paired Wilcox-test. Meanwhile, ISIRS can also give much higher predicted correlations than ENR, with the paired Wilcox-test (p-value = 0.0004297). It is concluded that the ISIRS method could identify some marginally weak features, and achieve better results than other methods.

**Fig. 4:**
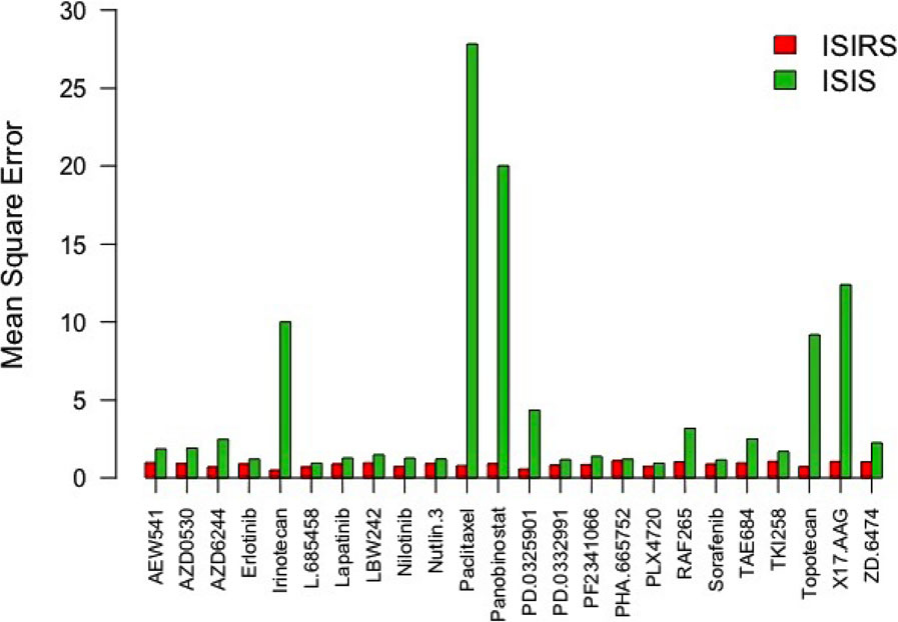
Comparison of ISIRS with ISIS method in drug sensitivity prediction. Mean square error between predicted (mean of 10 iterations of 10-fold cross-validations) and true drug sensitivities by ISIRS (red), ISIS (green)

**Fig. 5:**
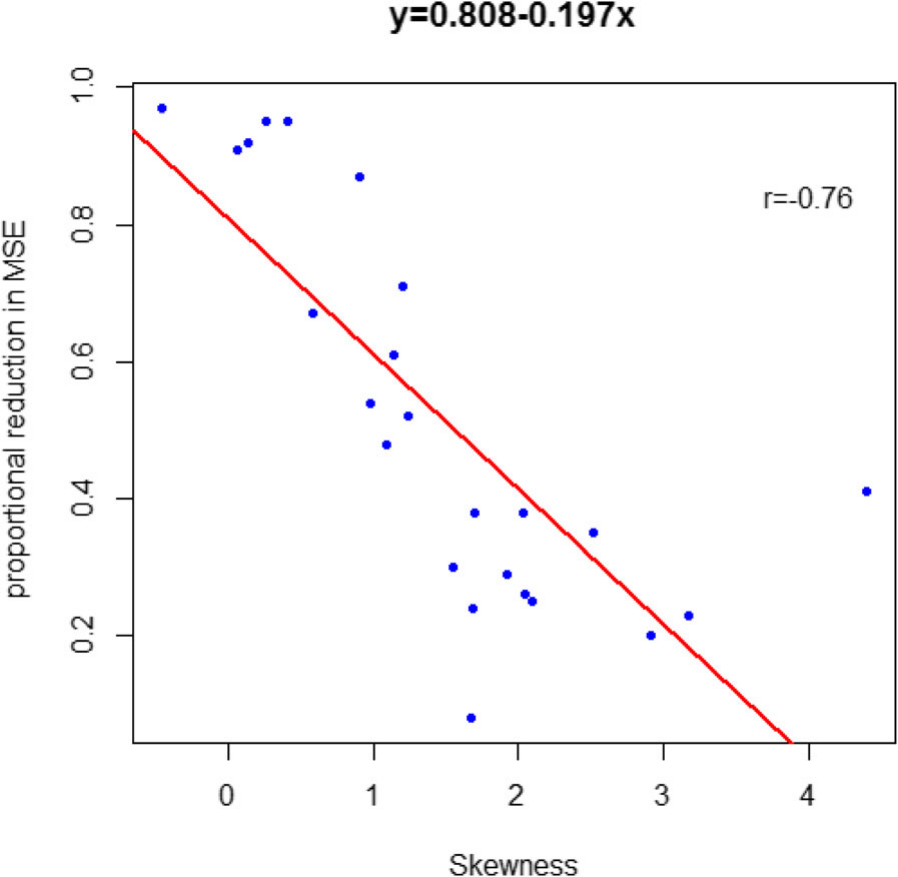
Scatter plots of the skewness and proportional reduction in MSE for all drugs

Besides the aforementioned criterion of PCC, we also calculated the mean squared errors (MSE) of the averaged predicted values from the 10-fold cross validation to assess the predicting performance of ISIRS, which are shown in Fig. 4 by a bar plot. From the bar plot, we can observe that all the MSE of ISIRS is lower than ISIS with the Wilcox-test (*p*-value = 5.96e-08). And we can clearly observe that some of MSE from ISIS are much higher than those from ISIRS. By coincidence, we find that the distributions of the true drug sensitivity of these drugs are all close to normal distribution. We found that the relationship between the proportional reduction in MSE and skewness is negatively correlated (Fig. 5). It is well known that when the data distribution is gaussian distributed, the results will be more reliable. Therefore, when the true drug sensitivity values follow gaussian distribution, the results of ISIRS will be better.

**Fig. 6:**
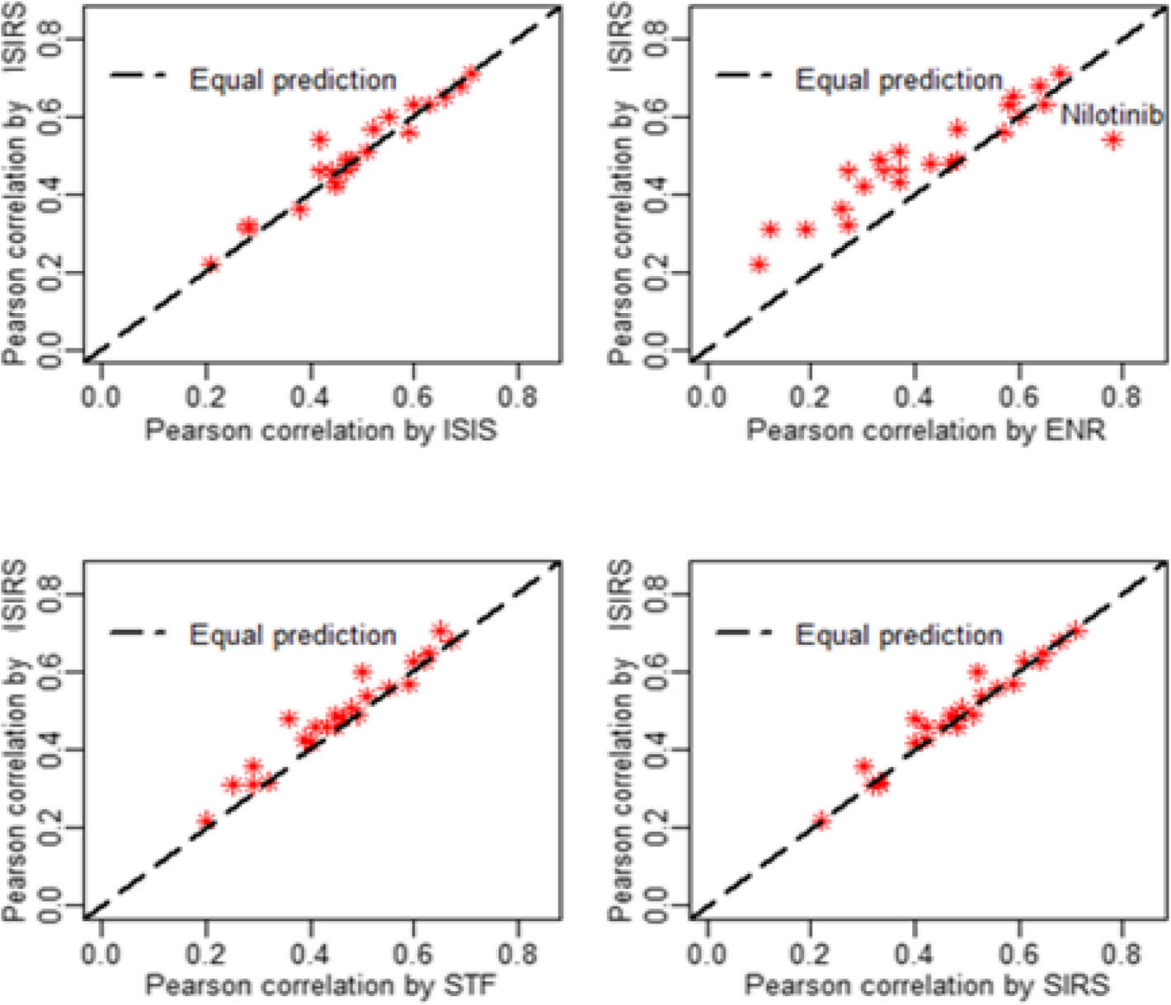
Consistence between correlations of true and predicted drug responses by elastic net, ISIS, STF, SIRS and ISIRS. Dotted line indicates equal predictions

Next, we analyzed the correlations of predicted drug responses between our ISIRS model and ENR, ISIS, STF and SIRS (Fig. 6). Our predictions were in good consistence with those by ENR model, given the overall correlation of 0.90. In particular, if we neglect the only one outlier, Nilotinib, the overall Pearson correlation will increase from 0.90 to 0.95. And our predictions were in great consistence with those by ISIS, STF and SIRS model, given the overall correlation of 0.97. From Fig. 6, we can see that ENR model brought a higher prediction correlation than ISRIS for drug Nilotinib. Because Nilotinib is a special compound for treating chronic myelogenous leukemia (CML) [28], which was successfully selected as the strongest feature for sensitivity of Nilotinib [8]. This top feature in ENR model dominated the model building and prediction, and brought a high prediction correlation. Except this unique outlier, ISIRS obtained higher predictive correlations by using fewer features than ENR.

We claimed that our ISIRS approach could efficiently eliminate redundancy among selected features. The mean redundancy score (MRS) [29], measured by the PCC and the mutual information (MI) [30], were used to evaluate the redundancy between identified features. In addition, we also implemented the simple top features (STF) method by ranking the features through the *ω*_*k*_, where the features in STF is the same as that in ISIRS. The MRSs for the 24 drugs through ISIRS and STF are listed in Table S3. If measured by PCC, the MRSs by ISIRS and STF are 0.2138 and 0.4420 respectively. It is suggested that the feature redundancy is significantly removed by ISIRS compared with STF (*p*-value = 2.384e-07 by paired Wilcox-test). Moreover, if measured by MI, the means of MIs by ISIRS and STF for the 24 drugs are 0.0830 and 0.2042 respectively, also showing significant difference by paired Wilcox-test (p-value = 4.712e-07). Details of the mutual information (MI) are shown in Table S4. All above results confirm that ISIRS could remove the redundancy between selected features.

## Conclusion

Predicting drug response from genomic data including gene expression, mutation status of genes and copy number alteration is a very fundamental problem in research of personalized medicine. In this paper, we applied an iterative sure independence ranking and screening (ISIRS) to select the features. Through cross validation on the CCLE dataset, we reported that our method could not only find numerous biomarkers which were reported in previous literatures, but also detect many marginally weak yet biologically important genomic features. These new detected features are shown to have strong combination effects on drug response. Based on the selected features, we performed lasso regression model to predict the drug response on CCLE dataset. The Pearson correlation coefficients between predicted and true drug sensitivities showed that our arithmetic got much higher correlations than ENR, ISIS, STF and SIRS. In the future, we plan to make an available web-server to implement the prediction method ISIRS in the paper.

## Supplementary information

**Additional file 1: Table S1** The selected variables and ω_k_ values for 24 drugs.

**Additional file 2: Table S2** Person correlation coefficients between true and predicted drug responses by ISIRS, ENR, ISIS, SIRS and STF.

**Additional file 3: Table S3** The mean redundancy score measured by PCC for the 24 drugs by ISIS, STF and ISIRS.

**Additional file 4: Table S4** The mean redundancy score measured by MI for the 24 drugs by ISIS, STF and ISIRS.

**Additional file 5: Fig. S1**: Pearson correlation coefficients of predicted and true drug sensitivities at different numbers of recruited features for the other 20 drugs.

## Data Availability

All data analyzed during this study are included in Dataset files. **Dataset file 1**: Drug response data in CCLE. **Dataset file 2**: Mutation status data in CCLE. **Dataset file 3**: Copy number alteration data in CCLE. **Dataset file 4**: Gene expression data in CCLE.

## Abbreviations

CCLE: Cancer Cell Line Encyclopedia
ISIRS: Iterative sure independent ranking and screening
ALL: Acute lymphoblastic leukemia
ISIS: Iterative sure independence screening
SIRS: Sure independent ranking and screening
STF: Simple top features
OLS: Ordinary least squares
PCC: Pearson correlation coefficient
MSE: Mean squared errors
MRS: Mean redundancy score
MI: Mutual information

## References

[1] Pal R, Berlow N, Haider S. Anticancer drug sensitivity analysis: an integrated approach applied to erlotinib sensitivity prediction in the ccle database. Proceedings 2012 IEEE International Workshop on Genomic Signal Processing and Statistics (GENSIPS). 2012;9–12.

[2] Staunton JE, Slonim DK, Coller HA, Tamayo P, Angelo MJ, Park J, Scherf U, Lee JK, Reinhold WO, Weinstein JN (2001). Chemosensitivity prediction by transcriptional profiling. Proc Natl Acad Sci U S A.

[3] Riddick G, Song H, Ahn S, Walling J, Borges-Rivera D, Zhang W, Fine HA (2011). Predicting in vitro drug sensitivity using random forests. Bioinformatics.

[4] Lee JK, Havaleshko DM, Cho H, Weinstein JN, Kaldjian EP, Karpovich J, Grimshaw A, Theodorescu D (2007). A strategy for predicting the chemosensitivity of human cancers and its application to drug discovery. Proc Natl Acad Sci U S A.

[5] Holleman A, Cheok MH, den Boer ML, Yang W, Veerman AJ, Kazemier KM, Pei D, Cheng C, Pui C-H, Relling MV (2004). Gene-expression patterns in drug-resistant acute lymphoblastic leukemia cells and response to treatment. N Engl J Med.

[6] Shen L, Kondo Y, Ahmed S, Boumber Y, Konishi K, Guo Y, Chen X, Vilaythong JN, Issa JP (2007). Drug sensitivity prediction by CpG island methylation profile in the NCI-60 cancer cell line panel. Cancer Res.

[7] Chen J, Peng H, Han G, Cai H, Cai J (2019). HOGMMNC: a higher order graph matching with multiple network constraints model for gene-drug regulatory modules identification. Bioinformatics.

[8] Wang Y, Wang Z, Xu J, Li J, Li S, Zhang M, Yang D (2018). Systematic identification of non-coding pharmacogenomic landscape in cancer. Nat Commun.

[9] Menden MP, Iorio F, Garnett M, McDermott U, Benes CH, Ballester PJ, Saez-Rodriguez J (2013). Machine learning prediction of cancer cell sensitivity to drugs based on genomic and chemical properties. PLoS One.

[10] Barretina J, Caponigro G, Stransky N, Venkatesan K, Margolin AA, Kim S, Wilson CJ, Lehar J, Kryukov GV, Sonkin D (2012). The Cancer cell line encyclopedia enables predictive modelling of anticancer drug sensitivity. Nature.

[11] Fang Y, Qin Y, Zhang N, Wang J, Wang H, Zheng X (2015). DISIS: prediction of drug response through an iterative sure independence screening. PLoS One.

[12] Fan J, Lv J (2008). Sure independence screening for ultrahigh dimensional feature space. J Royal Statistical Society: Series B (Statistical Methodology).

[13] Zhu L, Li L, Li R, Zhu L (2011). Model-free feature screening for ultrahigh dimensional data. J Am Stat Assoc.

[14] Sebaugh JL (2011). Guidelines for accurate EC50/IC50 estimation. Pharm Stat.

[15] Chen W, Lin H, Feng PM, Ding C, Zuo YC, Chou KC (2012). iNuc-PhysChem: a sequence-based predictor for identifying nucleosomes via physicochemical properties. PLoS One.

[16] Yao J, Chang C, Salmi ML, Hung YS, Loraine A, Roux SJ (2008). Genome-scale cluster analysis of replicated microarrays using shrinkage correlation coefficient. BMC bioinformatics.

[17] Zoppoli G, Regairaz M, Leo E, Reinhold WC, Varma S, Ballestrero A, Doroshow JH, Pommier Y (2012). Putative DNA/RNA helicase Schlafen-11 (SLFN11) sensitizes cancer cells to DNA-damaging agents. Proc Natl Acad Sci.

[18] Wu S, Guo Z, Hopkins CD, Wei N, Chu E, Wipf P, Schmitz JC (2015). Bis-cyclopropane analog of disorazole C1 is a microtubule-destabilizing agent active in ABCB1-overexpressing human colon cancer cells. Oncotarget.

[19] Martinelli G, Iacobucci I, Storlazzi CT, Vignetti M, Paoloni F, Cilloni D, Soverini S, Vitale A, Chiaretti S, Cimino G (2009). IKZF1 (Ikaros) deletions in BCR-ABL1–positive acute lymphoblastic leukemia are associated with short disease-free survival and high rate of cumulative incidence of relapse: a GIMEMA AL WP report. J Clin Oncol.

[20] Sheu JJ, Guan B, Choi JH, Lin A, Lee CH, Hsiao YT, Wang TL, Tsai FJ, Shih Ie M (2010). Rsf-1, a chromatin remodeling protein, induces DNA damage and promotes genomic instability. J Biol Chem.

[21] Kamei T, Matozaki T, Sakisaka T, Kodama A, Yokoyama S, Peng YF, Nakano K, Takaishi K, Takai Y (1999). Coendocytosis of cadherin and c-met coupled to disruption of cell-cell adhesion in MDCK cells--regulation by rho, Rac and Rab small G proteins. Oncogene.

[22] Yu MG, Zheng HY (2017). Acute myeloid leukemia: advancements in diagnosis and treatment. Chin Med J.

[23] Zhou YZB, Pache L, Chang M, Khodabakhshi AH, Tanaseichuk O, Benner C, Chanda SK (2019). Metascape provides a biologist-oriented resource for the analysis of systems-level datasets. Nat Commun.

[24] Kozak W, Dasko M, Maslyk M, Kubinski K, Rachon J, Demkowicz S (2015). Steroid Sulfatase inhibitors based on phosphate and Thiophosphate flavone analogs. Drug Dev Res.

[25] Yu R, Li Z, Zhang C, Song H, Deng M, Sun L, Xu L, Che X, Hu X, Qu X (2019). Elevated limb-bud and heart development (LBH) expression indicates poor prognosis and promotes gastric cancer cell proliferation and invasion via upregulating integrin/FAK/Akt pathway. PeerJ.

[26] Santidrian AF, Matsuno-Yagi A, Ritland M, Seo BB, LeBoeuf SE, Gay LJ, Yagi T, Felding-Habermann B (2013). Mitochondrial complex I activity and NAD+/NADH balance regulate breast cancer progression. J Clin Invest.

[27] Ghadban T, Jessen A, Reeh M, Dibbern JL, Mahner S, Mueller V, Wellner UF, Gungor C, Izbicki JR, Vashist YK (2016). In vitro study comparing the efficacy of the water-soluble HSP90 inhibitors, 17-AEPGA and 17-DMAG, with that of the nonwater-soluble HSP90 inhibitor, 17-AAG, in breast cancer cell lines. Int J Mol Med.

[28] DeRemer DL, Ustun C, Natarajan K (2008). Nilotinib: a second-generation tyrosine kinase inhibitor for the treatment of chronic myelogenous leukemia. Clin Ther.

[29] Ren X, Wang Y, Chen L, Zhang X-S, Jin Q (2012). EllipsoidFN: a tool for identifying a heterogeneous set of cancer biomarkers based on gene expressions. Nucleic Acids Res.

[30] Peng H, Long F, Ding C (2005). Feature selection based on mutual information criteria of max-dependency, max-relevance, and min-redundancy. IEEE Trans Pattern Anal Mach Intell.

