# Supplementary material for: Iterative sure independent ranking and screening for drug response prediction
Source: BMC Med Inform Decis Mak. 2020 Sep 22;20(Suppl 8):224. doi: 10.1186/s12911-020-01240-9 (PMC7507262; doi:10.1186/s12911-020-01240-9)
Supplement: Supplementary file 1 — Additional file 1: Table S1 The selected variables and ωk values for 24 drugs. [file 12911_2020_1240_MOESM1_ESM.pdf]

Table 1: The selected variables, ω_k values and the orders based on ω_k for 24 drugs.

**drugs**	**types of variable**	**variable names**	**ω_k values**	**Orders**
**X17.AAG**	gene expression	ZFP30	0.00684675	1
	gene expression	OGDHL	0.006503799	3
	gene expression	LOC100507372	0.006461819	4
	gene expression	TMEM130	0.006246967	5
	gene expression	ZNF420	0.006213836	6
	gene expression	RPUSD4	0.00534112	10
	gene expression	NFKB1	0.005213797	13
	gene expression	GFRA1	0.00521276	14
	gene expression	NQO1	0.005211578	15
	gene expression	MAP7D2	0.004968159	17
	gene expression	CTDSP1	0.004945059	18
	gene expression	CELF5	0.004938793	19
	gene expression	CLDN16	0.004907411	20
	gene expression	ABCE1	0.004764505	21
	gene expression	LBH	0.004675065	22
	gene expression	RAI2	0.004665078	24
	gene expression	LOC401317	0.004640632	25
	gene expression	SREBF1	0.004628072	26
	gene expression	ZNF253	0.00461629	27
	gene expression	C15orf57	0.004582086	28
	copy number	COL23A1	0.002579144	527
	copy number	FAM153C	0.003413631	183
	gene expression	**STARD9**	0.000280746	22240
	gene expression	ATAT1	0.000408026	16533

**X17.AAG**	gene expression	**MRPL21**	0.000235498	24961
	gene expression	**GNAS**	0.002193372	870
	gene expression	NFYA	0.000797453	7435
	copy number	**OSBPL3**	0.000613968	10798
	mutate site	**CYP27B1**	0.001090498	4420
	gene expression	**LOC286052**	0.001290337	3156
	gene expression	IKBKB	0.000294262	21541
	copy number	**PLEKHA2**	0.000869721	6470
	gene expression	**LRRC72**	0.001097946	4374
	gene expression	AARSD1	0.000665315	9679
	gene expression	**CEBPG**	0.000488768	13832
	gene expression	CSTA	0.003167469	229
	gene expression	**LOC96610**	0.002658439	492
	gene expression	STAR	0.00091354	5978
	gene expression	**ANKRD40**	0.001904532	1282
	gene expression	**SLC12A1**	0.000708885	8885
	gene expression	**RERG**	0.001547463	2137
	copy number	**UBE2MP1**	0.000974422	5380
**drugs**	**types of variable**	**variable names**	**ω_k values**	**Orders**
**AZD0530**	gene expression	MCU	0.008672302	1
	gene expression	ITGB6	0.007442834	2
	gene expression	**RSF1**	0.004116388	137
	gene expression	**THEM4**	0.005793965	21
**drugs**	types of variable	variable names	ω_k values	Orders
**Nutlin.3**	gene expression	DDB2	0.006887253	1
	gene expression	LOC100289026	0.006807505	2
	gene expression	SELPLG	0.00607898	3
	gene expression	WT1.AS	0.003380812	161

**drugs**	**types of variable**	**variable names**	**ω_k values**	**Orders**
	gene expression	CYR61	0.02665495	3
	gene expression	FHL2	0.0225542	11
	gene expression	ARSJ	0.02143725	19
	gene expression	GPC1	0.02136851	21
	gene expression	SH2D3C	0.02093588	24
	gene expression	EPB41	0.02070181	29
	copy number	**RAB24**	0.0119157	457
	gene expression	OSCP1	0.01147994	519
	gene expression	**C8orf49**	0.003730704	4708
	gene expression	POU5F2	0.002290497	7638
	gene expression	**C9orf79**	0.000631228	19899
**Panobinostat**	gene expression	**TAF3**	0.01083235	624
	gene expression	**C9orf41**	0.00998504	815
	gene expression	CCDC33	0.000303587	28380
	copy number	SH3RF2	0.0118797	461
	gene expression	**FBXO39**	0.001637549	10055
	gene expression	**APOC4**	0.00741414	1706
	gene expression	**OAZ1**	0.005934489	2375
	gene expression	**LOC284898**	0.001456015	11053
	copy number	C21orf99	0.000858747	16395
	gene expression	**NCOA7**	0.001076386	13932
	gene expression	**RBM26**	0.001643634	10030
	gene expression	ZNF233	0.001118719	13546
	copy number	POLR2G	0.003689342	4784
	gene expression	**FAM181A.AS1**	0.000372919	26085
	gene expression	**ANKRD20A11P**	0.004185025	3997
	gene expression	**LSM3**	0.00345996	5175
	mutate site	**HSPA4**	0.001476628	10936

**Panobinostat**	copy number	OR9Q1	0.002912368	6172
	gene expression	CTNND2	0.00355842	4993
	gene expression	ANKUB1	0.003162986	5708
	mutate site	**KRAS**	0.002595517	6849
	gene expression	LOC90499	0.002015301	8569
	copy number	MORC4	0.001143934	13322
	gene expression	**ADAMDEC1**	0.003775088	4644
	copy number	**CELA3A**	0.001276153	12260
	gene expression	**KIRREL3.AS3**	0.001425422	11231
	mutate site	RGL3	0.000426345	24645
	gene expression	EXOC3L4	0.001052649	14154
	mutate site	**ATF2**	0.000522678	22227
	gene expression	**LOC283484**	0.000377071	25971
	gene expression	FAM110A	0.000939656	15360
	gene expression	SLC25A18	0.000316238	27886
	gene expression	**LOC729810**	0.001645641	10019
	gene expression	**A4GNT**	0.001480826	10916
	mutate site	**AURKC**	0.001198252	12836
**drugs**	**types of variable**	**variable names**	**ω_k values**	**Orders**
**PD.0332991**	gene expression	TSEN34	0.01696563	1
	gene expression	PHLDB2	0.01629144	4
	gene expression	ARHGAP29	0.01619913	5
	gene expression	KDM2B	0.01564674	7
	gene expression	LINC00528	0.01520906	8
	gene expression	LOC645212	0.001630315	8260
	gene expression	GNGT2	0.000368181	23766
	gene expression	ADM	0.002384077	5566
	gene expression	**IGHV1.69**	0.000238502	29046
	gene expression	**C15orf57**	0.01095786	149

	gene expression	HR44	0.00486665	1824
	gene expression	LOC100129447	0.002784692	4628
**drugs**	**types of variable**	**variable names**	**ω_k values**	**Orders**
**PHA.665752**	gene expression	LAIR1	0.007789564	1
	gene expression	GMEB1	0.007334167	4
	gene expression	PIK3CG	0.007126214	6
	gene expression	DENND4A	0.006909803	7
	gene expression	FMNL1	0.006586998	10
	gene expression	PFN2	0.006323148	11
	gene expression	CDK9	0.006293744	12
	gene expression	ST8SIA4	0.006128969	14
	gene expression	SPCS2	0.006105696	15
	gene expression	FKBP5	0.006033228	17
	gene expression	CD63	0.000813741	9713
	gene expression	**INTU**	0.00049214	15824
	gene expression	GNS	0.000213199	27606
	gene expression	TMEM51	0.000836037	9368
	gene expression	MAFF	0.000472727	16380
	mutate site	KLF6	0.004307638	138
	gene expression	CLDN12	0.000748732	10674
	gene expression	CD1C	0.000144086	32147
	gene expression	ARHGEF40	0.000298421	23007
	gene expression	TYRO3	0.000410607	18302
	mutate site	**RHOA**	0.002480424	1031
	gene expression	**MTUS2**	0.000289833	23433
	gene expression	**CCDC67**	0.000569812	13911
	gene expression	**GPR20**	0.001417138	4012
	gene expression	ENAH	0.001106572	6209
	gene expression	PSD	0.000365684	20068

	gene expression	**CFC1B**	0.000941158	7950
	gene expression	**PLLP**	0.001166759	5685
**drugs**	**types of variable**	**variable names**	**ω_k values**	**Orders**
**RAF265**	gene expression	SH2B3	0.008471486	1
	gene expression	KIAA0090	0.007645775	2
	gene expression	C20orf197	0.007241993	3
	gene expression	EXOSC9	0.00717424	4
	gene expression	C2orf27A	0.007152362	5
	gene expression	FCER1G	0.007000229	6
	gene expression	LOC553103	0.006988862	7
	gene expression	GNPTAB	0.006816412	9
	gene expression	ZNF549	0.006782737	10
	gene expression	HIST1H2AK	0.006780521	11
	gene expression	HMG20A	0.006678909	13
	gene expression	ZEB2	0.006658216	15
	gene expression	C1orf174	0.006532221	16
	gene expression	CMTM3	0.006390683	17
	gene expression	LST1	0.00634825	18
	gene expression	ABHD11	0.006341112	20
	gene expression	ZNF185	0.000491082	20194
	gene expression	LGALSL	0.001092515	9905
	gene expression	**CYB5R2**	0.001073472	10091
	gene expression	**CRB1**	0.004292874	316
	gene expression	CEP41	0.001820064	4651
	gene expression	C15orf56	0.001546391	6076
	gene expression	TEAD4	0.002612189	2054
	gene expression	MKNK1	0.001678675	5328
	gene expression	**SLC16A7**	0.002286759	2900
	gene expression	MST1R	0.001403322	7057

	gene expression	TSPO	0.002810581	1682
	gene expression	FAM110C	0.001426534	6879
	gene expression	CADM1	0.001623065	5638
	gene expression	**CKAP5**	0.000338794	25424
**drugs**	**types of variable**	**variable names**	**ω_k values**	**Orders**
	gene expression	SELPLG	0.009547407	1
	gene expression	CORO7	0.008387034	2
	gene expression	RPL3	0.008115612	3
	gene expression	LST1	0.008046871	4
	gene expression	P2RX1	0.008032435	5
	gene expression	BCLAF1	0.007942542	6
	gene expression	ZNF496	0.007740382	8
	gene expression	MON2	0.007603915	9
	gene expression	ZCCHC11	0.007032029	14
	gene expression	**WAS**	0.003545155	669
	gene expression	NCKAP1L	0.001695446	3719
	gene expression	CLECL1	0.002390449	1917
	gene expression	**KIAA0748**	0.002335341	1998
**Sorafenib**	gene expression	CD37	0.002595914	1603
	gene expression	PTPRC	0.004238815	341
	gene expression	BTK	0.001997196	2690
	gene expression	CD52	0.001606529	4097
	gene expression	**ESCO2**	0.0028293	1300
	gene expression	PIM1	0.000712417	11841
	gene expression	**PRSS21**	0.00067962	12441
	copy number	**LMO7**	0.001084335	7337
	gene expression	**SNX1**	0.002312852	2046
**drugs**	**types of variable**	**variable names**	**ω_k values**	**Orders**
	gene expression	ARHGAP19	0.02181722	1

**Topotecan**	gene expression	PPIC	0.02146578	2
	gene expression	CD63	0.02085354	3
	gene expression	SLFN11	0.01905921	8
	gene expression	FTH1P5	0.01875109	10
	gene expression	TAF5	0.01817035	11
	gene expression	HCLS1	0.01804345	12
	gene expression	RFXAP	0.01791885	13
	gene expression	CD276	0.01783925	14
	gene expression	FAM114A1	0.01762156	15
	gene expression	PTTG1IP	0.0172761	18
	gene expression	AGPAT5	0.01720362	20
	gene expression	ADAT2	0.01716662	21
	gene expression	IKZF1	0.01714461	22
	gene expression	CRB1	0.000638364	16932
	gene expression	LOC145837	0.002974631	5016
	gene expression	NKAIN2	0.000231547	28968
	gene expression	LGSN	0.003449251	4199
	copy number	SF3A2	0.01542578	50
	gene expression	RASD1	0.004613385	2825
	gene expression	ZNF630	0.004309123	3151
	gene expression	**ZNF736**	0.001787286	8405
	gene expression	THG1L	0.008848666	732
	gene expression	**PPP1R3F**	0.005055742	2465
	gene expression	**CADM1**	0.002014186	7567
	gene expression	RPL36	0.01013104	469
	gene expression	**KCNE4**	0.003576733	4025
	gene expression	C1orf182	0.006072891	1761
	gene expression	ZNF581	0.00562591	2034

	copy number	GPR89A	0.001216603	11372
	gene expression	MFAP2	0.000452763	20582
	gene expression	**LOC389247**	0.000713216	15894
	gene expression	**CERK**	0.002068167	7382
	gene expression	**CCDC126**	0.000125829	34943
**drugs**	**types of variable**	**variable names**	**ω_k values**	**Orders**
TKI258	gene expression	MYO5B	0.009777198	1
	gene expression	PAICS	0.009211638	4
	gene expression	SEPT6	0.009094142	5
	copy number	STX16	0.008883579	7
	copy number	ARFRP1	0.00874152	9
	gene expression	KIAA1671	0.00868668	11
	gene expression	CTDSPL2	0.008679252	12
	gene expression	FAM193A	0.008653145	13
	gene expression	MRPS27	0.008564396	19
	gene expression	DYM	0.008422764	22
	gene expression	SLC48A1	0.000621305	17494
	gene expression	C1orf233	0.001302883	8654
	gene expression	POLR2J2	0.000883022	12965
	gene expression	ARHGEF19	0.000349013	25041
	gene expression	ANXA4	0.001061918	10754
	gene expression	SORBS3	0.001872148	5231
	gene expression	PRKCQ	0.005926937	278
	gene expression	TSPAN32	0.005142701	504
	gene expression	NBPF10	0.000704447	15861
	gene expression	CADM1	0.000724375	15488
	gene expression	HNRNPU.AS1	0.001619634	6531
	gene expression	NFE2	0.005673534	339
	gene expression	FZD6	0.000934606	12259

TKI258	gene expression	SOX15	0.000126107	35091
	gene expression	ZNF673	0.000168755	32997
	gene expression	**CD1C**	0.000919566	12466
	gene expression	PAX5	0.00101613	11286
	copy number	RASA4	0.00304742	2143
	gene expression	WT1.AS	0.002993522	2234
	gene expression	TPRG1L	0.000886686	12911
	gene expression	POLA1	0.001101249	10378
	gene expression	LOC728978	0.000498604	20518
	gene expression	LOC645638	0.001503013	7205
	gene expression	RUNX1T1	0.000149809	33955
	copy number	**LOC100287704**	0.000296816	26974
	gene expression	**SDS**	0.003317895	1791
**drugs**	**types of variable**	**variable names**	**ω_k values**	**Orders**
Irinotecan	gene expression	ARHGAP19	0.03374421	1
	gene expression	PPIC	0.02745005	2
	gene expression	SLFN11	0.02540681	5
	gene expression	**MAP3K4**	0.01464075	322
**drugs**	**types of variable**	**variable names**	**ω_k values**	**Orders**
Erlotinib	gene expression	SYTL1	0.01241243	1
	gene expression	PTPN6	0.01176568	2
	gene expression	STXBP2	0.01084681	3
	gene expression	EVPL	0.01048609	5
	gene expression	PAK6	0.01018985	6
	gene expression	FGFBP1	0.01002264	7
	gene expression	PRRG4	0.009929664	8
	gene expression	CDH3	0.009799539	9
	gene expression	FAM83B	0.009720856	12
	gene expression	TSTD1	0.009627447	13

**Erlotinib**	gene expression	CORO2A	0.009536388	14
	gene expression	PRSS22	0.00369016	580
	gene expression	C1orf172	0.006224979	125
	gene expression	CLDN4	0.004152501	444
	gene expression	**ABHD11**	0.000347452	19952
	gene expression	**C6orf132**	0.002785865	1066
	copy number	BMP4	0.000326816	20937
	copy number	EAPP	0.000241382	25759
	copy number	HEATR5A	0.000342942	20156
	copy number	**EIF3L**	0.000921004	7031
	gene expression	EML1	0.001720455	2553
	gene expression	**GNA15**	0.003870455	512
	gene expression	**ADAMTS1**	0.000741234	9291
	gene expression	**C3orf49**	0.000123274	33852
**drugs**	**types of variable**	**variable names**	**ω_k values**	**Orders**
**Nilotinib**	gene expression	SELPLG	0.01391462	1
	gene expression	ELF2	0.01217276	2
	gene expression	ITGA3	0.01130214	3
	gene expression	SRBD1	0.0110457	4
	gene expression	ITM2A	0.01029447	6
	gene expression	ARHGAP29	0.01029182	7
	gene expression	AHNAK2	0.0101043	8
	gene expression	WAS	0.004594691	778
	gene expression	NCKAP1L	0.002645839	2812
	gene expression	FKBP9	0.005084454	539

**Nilotinib**	**gene expression**	**IKZF1**	**0.007696422**	**80**
	gene expression	**RAB13**	0.003216763	1916
	gene expression	TRAF3IP3	0.006464476	1440
	gene expression	**RASAL3**	0.003624263	209

**drugs**	**types of variable**	**variable names**	**ω_k values**	**Orders**
**AEW541**	gene expression	MRPL50	0.009101363	1
	gene expression	ACVR2B	0.007627941	2
	gene expression	IGF1R	0.007272641	3
	gene expression	SNAPC1	0.006602928	5
	gene expression	ARID3A	0.006602885	6
	gene expression	IQGAP2	0.006551503	7
	gene expression	TMEM101	0.006294037	9
	gene expression	ALCAM	0.006166287	10
	gene expression	HIF1A	0.006047309	11
	gene expression	MT1X	0.005611697	14
	gene expression	TAF13	0.005286462	16
	gene expression	BNIP3	0.005214512	17
	gene expression	LIME1	0.005202262	18
	gene expression	TRIM6	0.005186779	20
	gene expression	PLEKHM3	0.005152764	21
	gene expression	SLC44A1	0.005132065	22
	gene expression	RAB23	0.005082779	23
	gene expression	ANKRD46	0.005043975	24
	gene expression	C11orf2	0.00502922	25

	gene expression	C14orf93	0.005019807	26
	gene expression	PEA15	0.004980631	27
**AEW541**	gene expression	SBNO1	0.004965029	28
	gene expression	**MGC70870**	0.000761319	8285
	gene expression	CD69	0.000268272	23650
	gene expression	AMIGO2	0.000964648	5832
	gene expression	**PPM1K**	0.00066457	9938
	gene expression	**CAV1**	0.000640993	10399
	gene expression	**GPR98**	0.003085203	276
	gene expression	**PROSER1**	0.000547645	12530
	gene expression	LOC155060	0.000651833	10187
	gene expression	**SNN**	0.002656779	451
	copy number	PARP8	0.000728867	8795
	gene expression	**HNRNPU.AS1**	0.000243931	25245
	gene expression	**SOAT1**	0.004607817	45
	gene expression	METTL8	0.0001435	32631
	copy number	**PLK2**	0.000680501	9567
	gene expression	C11orf46	0.001117664	4552
	gene expression	ORC2	0.000253314	24633
	gene expression	PARP15	0.000117795	34496
	gene expression	**TRAP1**	0.001765377	1768
	gene expression	**PTENP1**	0.002620755	468
	gene expression	NANOG	0.002878616	359
	gene expression	C12orf54	0.000871462	6845

	gene expression	**LOC100128175**	0.000978278	5696

**drugs**	types of variable	variable names	ω_k values	Orders
**AZD6244**	gene expression	LYZ	0.01585372	1
	gene expression	RNF125	0.01558548	2
	gene expression	SPRY2	0.01373113	4
	gene expression	ETV4	0.01369916	5
	gene expression	HSD17B11	0.01206317	6
	mutate site	BRAF	0.01197284	7
	gene expression	CAPN3	0.01014465	9
	gene expression	C7orf70	0.009640504	13
	gene expression	OSBPL3	0.009360076	18
	gene expression	FTSJ1	0.002701939	1262
	gene expression	KLF17	0.004353208	368
	gene expression	MYLK	0.004269132	393
	gene expression	GJB5	0.002736346	1220
	gene expression	HCCS	0.003099534	906
	gene expression	MFGE8	0.001228909	5505
	gene expression	CDR2	0.004304073	382
	mutate site	**NRAS**	0.005036108	234
	gene expression	LOC284837	0.000755691	10384
	gene expression	MIR205HG	0.000613139	13206
	gene expression	VAPA	0.002502449	1510
	gene expression	**ACY1**	0.001618191	3528
	gene expression	CHST3	0.001987061	2439

	gene expression	CEP95	0.000745267	10563
	gene expression	FCGR2C	0.004166507	419
	gene expression	C12orf23	0.004318203	377
	gene expression	LY6D	0.001437106	4321
	gene expression	**LOC645638**	0.003209662	852
	gene expression	**HMGB3**	0.002239599	1913
**drugs**	types of variable	variable names	$\boldsymbol{\omega_k}$ values	Orders
	gene expression	TAF5	0.01770644	1
	gene expression	HCLS1	0.01654008	4
	gene expression	SSRP1	0.01643228	5
	gene expression	ATP1B1	0.01563534	8
	gene expression	CTDSPL2	0.0152119	10
	gene expression	DUT	0.01518339	11
	gene expression	SNRPA	0.01491433	12
	mutate site	ADAT2	0.01449369	14
	gene expression	RPUSD2	0.01422681	18
	gene expression	ANP32B	0.01397689	20
	gene expression	FAM98B	0.01381943	22
	gene expression	POLE3	0.01377259	24
**Paclitaxel**	copy number	**ABCB1**	0.003783099	3032
	gene expression	HOXC10	0.000273974	28340
	gene expression	SERPINB3	0.001688738	7656
	gene expression	MACROD2	0.003863066	2937
	gene expression	JUNB	0.00047435	21632

Paclitaxel	gene expression	PSMG3	0.00284761	4243
	gene expression	LYPD6B	0.000173506	32991
	gene expression	SLC7A11	0.000243823	29647
	gene expression	PDRG1	0.001823519	7127
	gene expression	NLK	0.000131754	35093
	gene expression	SREBF1	0.001693414	7638
	gene expression	SH3PXD2B	0.001082691	11728
	gene expression	CLRN1.AS1	0.00053154	20161
	gene expression	STEAP3	0.00125464	10264
	gene expression	PPAP2C	0.002735148	4481
	gene expression	SERPINB4	0.001085865	11687
	gene expression	TPRXL	0.001353572	9518
	gene expression	ZC3H10	0.000624181	18048
	gene expression	ARTN	0.001100614	11559
	copy number	**PALM2**	0.000466584	21842
	gene expression	PTHLH	0.000506969	20744
	gene expression	**HIST1H3D**	0.000555752	19561
	gene expression	**ZNF655**	0.003900654	2894
	copy number	TEF	0.001426023	9042
**drugs**	types of variable	variable names	ω_k values	Orders
**PD.0325901**	gene expression	DUSP6	0.01837579	1
	gene expression	ETV4	0.01822278	2
	gene expression	RNF125	0.0174631	3
	gene expression	SPRY2	0.01711567	4

	gene expression	LYZ	0.01641221	5
	gene expression	HSD17B11	0.01400261	6
	gene expression	SPRY4.IT1	0.01184283	9
	mutate site	BRAF	0.01159654	10
	gene expression	GJB1	0.01134417	11
	gene expression	LPCAT2	0.01126175	12
	gene expression	SH3TC2	0.0112032	13
	gene expression	RPL4	0.003548828	846
	gene expression	WNK3	0.004970973	367
	gene expression	**PYCARD**	0.005798888	232
	gene expression	**DDB2**	0.006327649	184
**PD.0325901**	gene expression	EML1	0.008670816	49
	gene expression	ZFAND6	0.002323334	2234
	mutate site	**NRAS**	0.004823687	396
	gene expression	MFGE8	0.001124257	7062
	gene expression	USP27X	0.000101818	36243
**drugs**	types of variable	variable names	ω_k values	Orders
	gene expression	DDR1	0.01237046	1
	gene expression	ELF2	0.01140558	3
**PF2341066**	gene expression	PTPN7	0.01128657	4
	gene expression	ATP1B1	0.01115035	5
	gene expression	CBFA2T3	0.01045103	7
	gene expression	TPD52L1	0.01041116	9
	gene expression	GHRLOS2	0.01030373	11

**PF2341066**	gene expression	BLK	0.000716443	14074
	gene expression	FCRLA	0.000281533	25488
	gene expression	**HGF**	0.007601821	116
	gene expression	CD1C	0.000277695	25691
	gene expression	LOC100129447	0.000870439	12073
	gene expression	FCGR2B	0.000281371	25507
	gene expression	C9orf125	0.000720899	14002
	gene expression	MS4A1	0.000814164	12731
	gene expression	CD79A	0.001064358	10141
	gene expression	SEPT1	0.002006434	5056
	gene expression	BLNK	0.000372375	21910
**drugs**	types of variable	variable names	ω_k values	Orders
**PLX4720**	mutate site	BRAF	0.01106687	1
	gene expression	SPRYD5	0.01058307	3
	gene expression	BCL2A1	0.00927414	7
	gene expression	RXRG	0.009239536	8
	gene expression	MAD1L1	0.009179018	9
	gene expression	CAPN3	0.009165298	10
	gene expression	**RNF32**	0.002908575	643
	mutate site	**OR51A2**	0.001924899	1940
	gene expression	PFDN5	0.004028295	229
	gene expression	ZNF736	0.005642587	73
	mutate site	ANKK1	0.000692925	10961
	gene expression	LOC100505986	0.00517948	108

	gene expression	**IQGAP2**	0.002206272	1421
	mutate site	ATP8B1	0.001385183	3945
	gene expression	FZD9	0.000377422	19813
	gene expression	VAPA	0.000714562	10579
	mutate site	PI4KA	0.001163284	5341
	gene expression	FRG1B	0.001605452	2946
**drugs**	types of variable	variable names	ω_k values	Orders
**L.685458**	gene expression	MSL2	0.01316814	1
	gene expression	PIK3CG	0.01220304	3
	gene expression	CTSL1	0.01181257	4
	gene expression	PRSS23	0.01168344	5
	gene expression	NTN4	0.01161437	6
	gene expression	SELPLG	0.0115657	7
	gene expression	MRPL50	0.01146797	8
	gene expression	CD63	0.003981762	1550
	gene expression	FAM114A1	0.01129175	10
	copy number	**PDXDC1**	0.001856444	5058
	gene expression	TIMP2	0.002756735	2994
	gene expression	CTGF	0.004991939	938
**drugs**	types of variable	variable names	ω_k values	Orders
**Lapatinib**	gene expression	GRB7	0.0149368	7
	gene expression	SYNGR2	0.01444461	10
	gene expression	SYTL1	0.0143539	11
	gene expression	EPN3	0.01422402	13

**Lapatinib**	gene expression	S100A14	0.01415335	14
	gene expression	AP1G2	0.01410741	15
	gene expression	PRSS16	0.01391556	19
	gene expression	**C11orf52**	0.007565106	235
	gene expression	**RAB17**	0.002452448	2630
	gene expression	CNKSR1	0.01135515	67
	gene expression	QSOX1	0.001775032	4386
	gene expression	**CHSY1**	0.002290354	2963
	gene expression	**NRAS**	0.000297523	24161
	gene expression	C6orf132	0.007612619	230
	gene expression	ARHGEF5	0.00730768	260
	gene expression	PLEKHG6	0.006255281	392
	gene expression	ZNF70	0.001391762	6278
	gene expression	CCDC64B	0.01205996	53
	copy number	OSBPL3	0.000677292	13584
	copy number	MEOX2	0.000455631	18551
	copy number	AGR2	0.000541771	16378
	gene expression	DCTN1	0.000502621	17300
	gene expression	PHF3	0.000260339	25912
	mutate site	PRKD1	0.001796272	4307
	gene expression	KIAA1407	0.000588103	15374
	gene expression	UCN	0.001129629	8072
	copy number	**LCTL**	0.00048175	17835
	gene expression	PPP2CB	0.000146667	32494

	gene expression	LOC100507404	0.000434096	19191
	gene expression	RRNAD1	0.002094358	3437
**drugs**	types of variable	variable names	ω_k values	Orders
**LBW242**	copy number	AHI1	0.005451275	1
	gene expression	RIPK1	0.005295163	2
	gene expression	KRTAP4.6	0.004331674	22
	gene expression	**CA5BP1**	0.001498003	1589
**drugs**	types of variable	variable names	ω_k values	Orders
**ZD.6474**	gene expression	MBTPS2	0.006868705	1
	gene expression	PRDX4	0.006749172	2
	gene expression	REL	0.006691356	3
	gene expression	SNAP25	0.006385267	4
	gene expression	STXBP1	0.006349345	5
	gene expression	SH3BP1	0.005970604	8
	gene expression	APOO	0.00595544	9
	gene expression	TET3	0.005944356	10
	gene expression	PGAP2	0.005728606	12
	gene expression	C16orf45	0.005661189	13
	gene expression	LPPR2	0.005174268	15
	gene expression	PELI1	0.005171833	16
	gene expression	ENPP1	0.005133118	18
	gene expression	IFNGR1	0.005072705	19
	gene expression	KLF9	0.005071844	20
	gene expression	**PABPC4L**	0.00225666	731

	gene expression	PNMAL1	0.000197403	26834
	gene expression	CMTM3	0.000239776	23856
	gene expression	**LOC440993**	0.000322461	19064
	gene expression	**FAT4**	0.000261408	22499
	gene expression	FAM57A	0.000394423	15872
**ZD.6474**	gene expression	KIAA1324	0.003630657	113
	gene expression	C9orf21	0.000352074	17638
	gene expression	RRNAD1	0.00022549	24815
	gene expression	CRISPLD1	0.000647956	9068
	gene expression	RAB26	0.00184278	1276
	gene expression	ROBO1	0.000651854	8996
	copy number	**VSTM2A**	0.001511152	2083
	gene expression	**LARGE**	0.000135177	31881
	gene expression	**ALPL**	0.000237698	23989
**drugs**	types of variable	variable names	ω_k values	Orders
	gene expression	ARID3A	0.01175131	1
	gene expression	HSPC072	0.009225723	2
**TAE684**	gene expression	MRPL50	0.008772887	3
	gene expression	SELPLG	0.0087369	4
	gene expression	EEF1B2	0.008448708	5
	gene expression	PRKX	0.007889999	6
	gene expression	CTDSPL2	0.007331765	11
	gene expression	BPIFB6	0.007272607	12
	gene expression	KDM5A	0.007195533	13

**TAE684**	gene expression	GMFG	0.007019398	14
	gene expression	SLC38A5	0.006936109	16
	gene expression	ALS2CR8	0.00680752	19
	gene expression	MT1X	0.006785699	21
	gene expression	MAP2K7	0.006714355	23
	gene expression	SLC10A7	0.006691924	24
	gene expression	TNFRSF12A	0.006679462	25
	gene expression	CD200R1	0.000314877	24926
	gene expression	CXorf59	0.001097234	8013
	copy number	**HDHD1A**	0.002838563	1287
	gene expression	**CD69**	0.001016821	8835
	gene expression	TGFBR1	0.001155932	7418
	gene expression	PPM1K	0.000986686	9215
	gene expression	GNMT	0.001631193	4207
	gene expression	MGC70870	0.00116187	7367
	gene expression	**PRC1**	0.000568164	16521
	gene expression	**PTPN13**	0.002908417	1195
	gene expression	QRSL1	0.000301669	25547
	gene expression	AKR1E2	0.000500043	18235
	gene expression	WSB2	0.001676582	4021
	gene expression	LOC155060	0.001225899	6789
	gene expression	IMPACT	0.000362934	22908
	gene expression	TMEM117	0.001200777	7023
	gene expression	KDELR1	0.00118838	7118

	gene expression	KLRK1	0.000403099	21327
	gene expression	**SOGA2**	0.001204417	6999
	gene expression	**IL4R**	0.000832134	11339
	mutate site	**NBN**	0.000977913	9311
	gene expression	DGCR10	0.000530643	17421
