# Supplementary material for: Iterative sure independent ranking and screening for drug response prediction
Source: BMC Med Inform Decis Mak. 2020 Sep 22;20(Suppl 8):224. doi: 10.1186/s12911-020-01240-9 (PMC7507262; doi:10.1186/s12911-020-01240-9)
Supplement: Supplementary file 2 — Additional file 2: Table S2 Person correlation coefficients between true and predicted drug responses by ISIRS, ENR, ISIS, SIRS and STF. [file 12911_2020_1240_MOESM2_ESM.pdf]

Table S2: The Person correlation coefficients between real and predicted drug responses by ISIRS, ENR, ISIS，SIRS and STF.

**methods**	**17.AAG**	**AEW541**	**AZD0530**	**AZD6244**	**Erlotinib**	**Irinotecan**
**ISIS**	0.47	0.47	0.28	0.63	0.45	0.71
**ENR**	0.43	0.33	0.19	0.58	0.3	0.68
**STF**	0.36	0.45	0.25	0.62	0.4	0.65
**ISIRS**	0.48	0.49	0.31	0.63	0.42	0.71
**SIRS**	0.4	0.47	0.32	0.64	0.4	0.71

**methods**	**L.685458**	**LBW242**	**Lapatinib**	**Nilotinib**	**Nutlin.3**	**PD.0325901**
**ISIS**	0.52	0.21	0.48	0.42	0.28	0.69
**ENR**	0.48	0.1	0.47	0.78	0.12	0.64
**STF**	0.59	0.2	0.46	0.51	0.29	0.67
**ISIRS**	0.57	0.22	0.48	0.54	0.31	0.68
**SIRS**	0.59	0.22	0.48	0.53	0.33	0.68

**methods**	**PD.0332991**	**PF2341066**	**PHA.665752**	**PLX4720**	**Paclitaxel**	**Panobinostat**
**ISIS**	0.48	0.44	0.28	0.59	0.55	0.6
**ENR**	0.48	0.37	0.27	0.57	0.6	0.65
**STF**	0.49	0.43	0.32	0.55	0.5	0.6
**ISIRS**	0.49	0.46	0.32	0.46	0.6	0.63
**SIRS**	0.51	0.45	0.33	0.56	0.52	0.61

**methods**	**RAF265**	**Sorafenib**	**TAE684**	**TKI258**	**Topotecan**	**ZD.6474**
**ISIS**	0.45	0.47	0.51	0.42	0.66	0.38
**ENR**	0.37	0.27	0.37	0.34	0.59	0.26
**STF**	0.39	0.45	0.48	0.41	0.63	0.29
**ISIRS**	0.43	0.46	0.51	0.46	0.65	0.36
**SIRS**	0.42	0.48	0.49	0.42	0.65	0.3
