# Supplementary material for: Iterative sure independent ranking and screening for drug response prediction
Source: BMC Med Inform Decis Mak. 2020 Sep 22;20(Suppl 8):224. doi: 10.1186/s12911-020-01240-9 (PMC7507262; doi:10.1186/s12911-020-01240-9)
Supplement: Supplementary file 3 — Additional file 3: Table S3 The mean redundancy score measured by PCC for the 24 drugs by ISIS, STF and ISIRS. [file 12911_2020_1240_MOESM3_ESM.pdf]

Table S3: The mean redundancy score measured by PCC for the 24 drugs by ISIS, STF and ISIRS.

Methods	AEW541	AZD0530	AZD6244	Erlotinib	Irinotecan	L.685458
**ISIRS**	0.1609	0.1749	0.1177	0.3286	0.3454	0.391
**STF**	0.2659	0.5363	0.2772	0.5453	0.588	0.471
**ISIS**	0.2094	0.1252	0.1426	0.1658	0.2448	0.4042

Methods	Lapatinib	LBW242	Nilotinib	Nutlin.3	Paclitaxel	Panobinostat
**ISIRS**	0.239	0.0821	0.5312	0.151	0.1517	0.0765
**STF**	0.6824	0.536	0.4913	0.27	0.4511	0.6129
**ISIS**	0.3592	0.1477	0.1348	0.2262	0.2172	0.1045

Methods	PD.0332991	PD.0325901	PF2341066	PHA.665752	PLX4720	RAF265
**ISIRS**	0.274	0.165	0.3789	0.202	0.119	0.1647
**STF**	0.6497	0.2698	0.5691	0.4614	0.5998	0.2371
**ISIS**	0.2757	0.1326	0.0535	0.265	0.1298	0.1294

Methods	Sorafenib	TAE684	TKI258	Topotecan	17.AAG	ZD.6474
**ISIRS**	0.344	0.1536	0.159	0.1536	0.0929	0.1756
**STF**	0.3893	0.348	0.411	0.5108	0.148	0.2875
**ISIS**	0.2394	0.1364	0.2304	0.2179	0.169	0.1559
