# Supplementary material for: Iterative sure independent ranking and screening for drug response prediction
Source: BMC Med Inform Decis Mak. 2020 Sep 22;20(Suppl 8):224. doi: 10.1186/s12911-020-01240-9 (PMC7507262; doi:10.1186/s12911-020-01240-9)
Supplement: Supplementary file 4 — Additional file 4: Table S4 The mean redundancy score measured by MI for the 24 drugs by ISIS, STF and ISIRS. [file 12911_2020_1240_MOESM4_ESM.pdf]

Table S4: The mean redundancy score measured by MI for the 24 drugs by ISIS, STF and ISIRS.

Methods	AEW541	AZD0530	AZD6244	Erlotinib	Irinotecan	L.685458
ISIRS	0.065	0.072	0.051	0.149	0.118	0.152
STF	0.097	0.255	0.078	0.256	0.228	0.178
ISIS	0.07	0.0558	0.0534	0.083	0.1003	0.0957

Methods	Lapatinib	LBW242	Nilotinib	Nutlin.3	Paclitaxel	Panobinostat
ISIRS	0.113	0.046	0.215	0.07	0.06	0.045
STF	0.356	0.965	0.193	0.103	0.155	0.253
ISIS	0.1518	0.0448	0.0464	0.1045	0.0864	0.0579

Methods	PD.0332991	PD.0325901	PF2341066	PHA.665752	PLX4720	RAF265
ISIRS	0.099	0.06	0.089	0.073	0.036	0.067
STF	0.282	0.087	0.189	0.137	0.102	0.086
ISIS	0.1157	0.0502	0.0983	0.0667	0.0373	0.0509

Methods	Sorafenib	TAE684	TKI258	Topotecan	17.AAG	ZD.6474
ISIRS	0.102	0.059	0.06	0.074	0.05	0.066
STF	0.11	0.109	0.342	0.184	0.06	0.096
ISIS	0.0643	0.0559	0.0934	0.0883	0.0707	0.0656
