# Supplementary figures and images for: Iterative sure independent ranking and screening for drug response prediction
Source: BMC Med Inform Decis Mak. 2020 Sep 22;20(Suppl 8):224. doi: 10.1186/s12911-020-01240-9 (PMC7507262; doi:10.1186/s12911-020-01240-9)

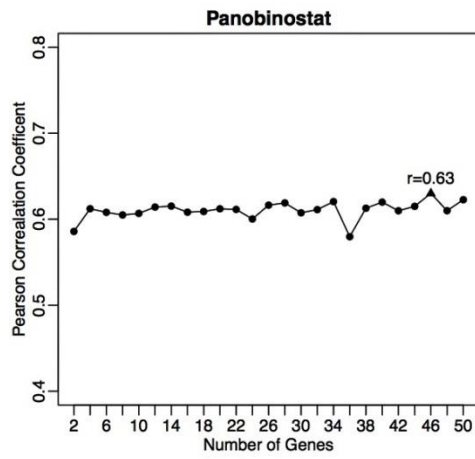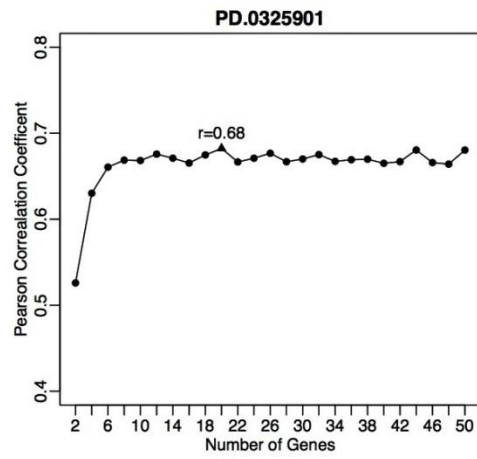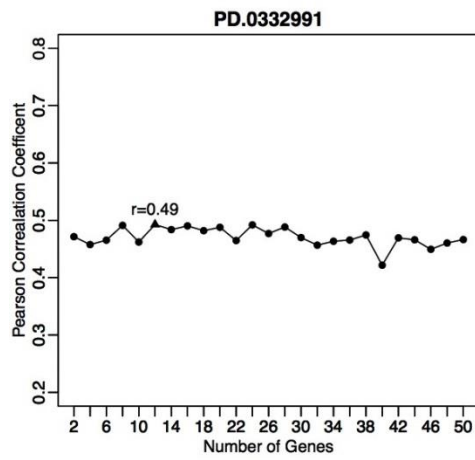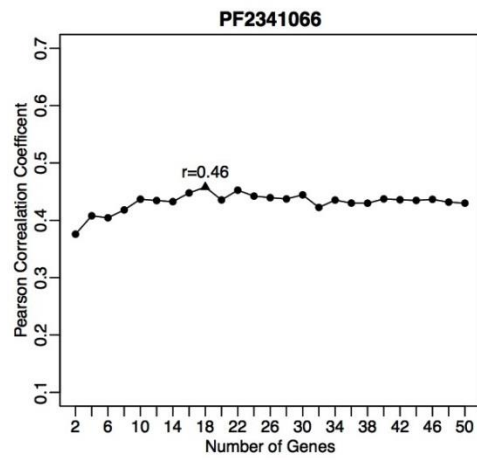

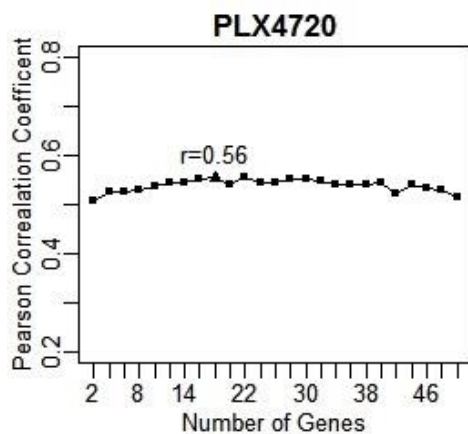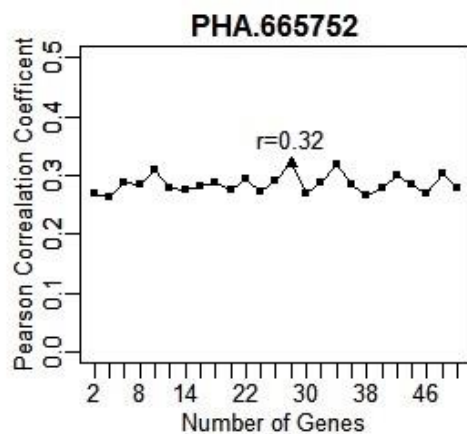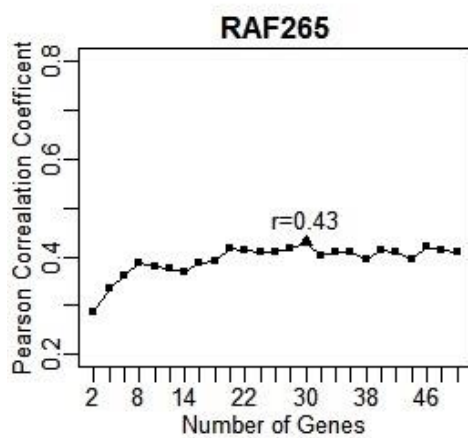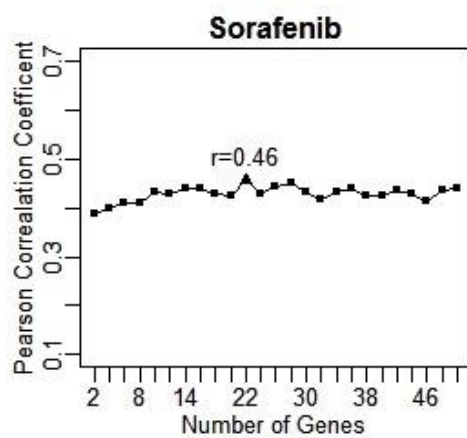

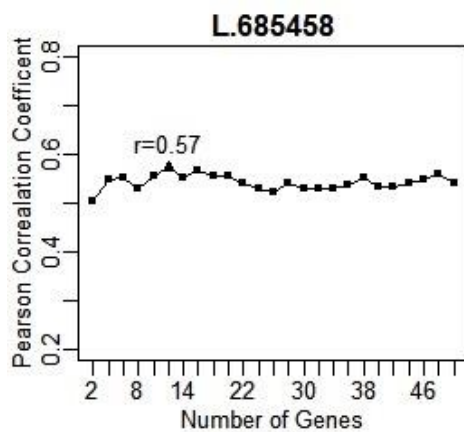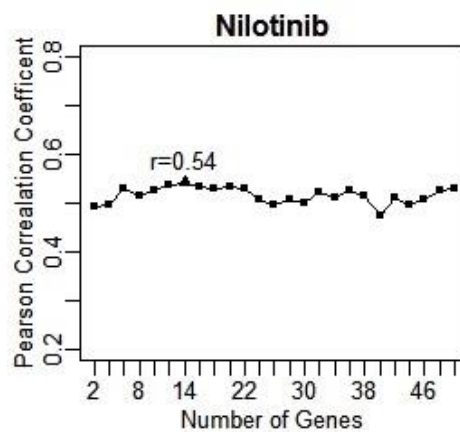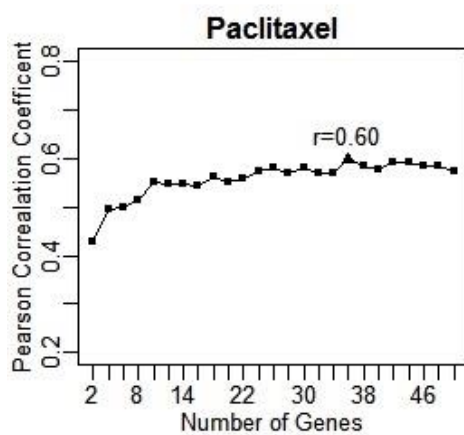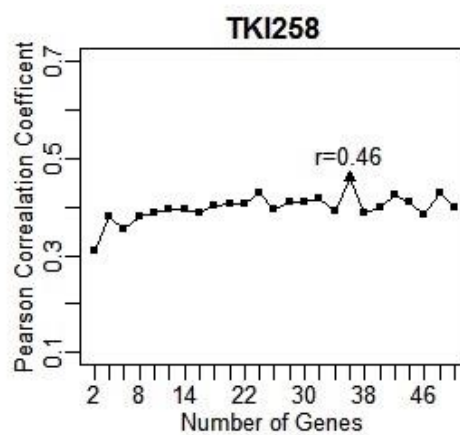

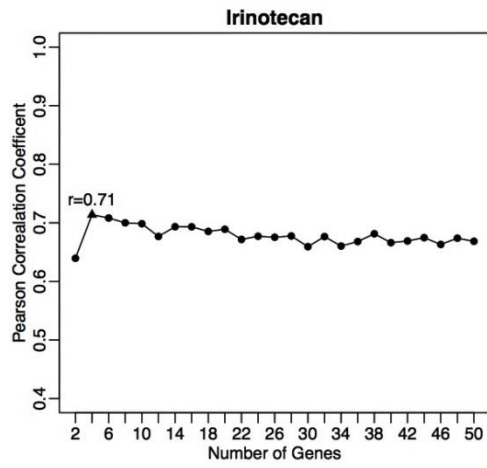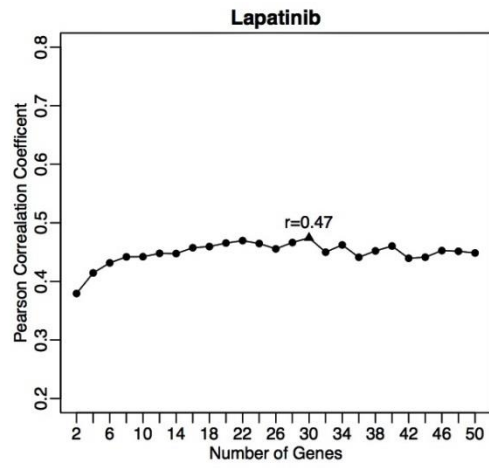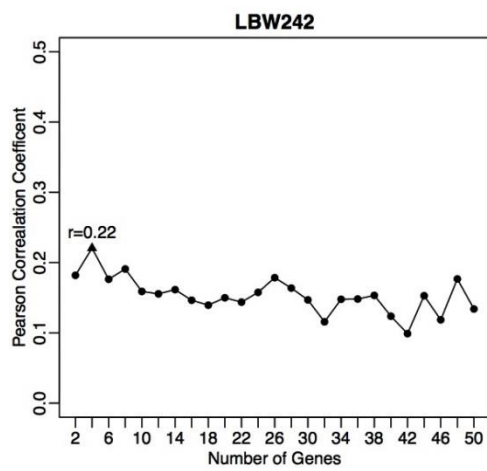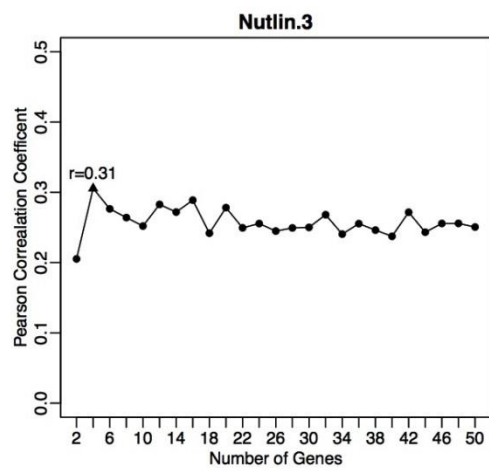

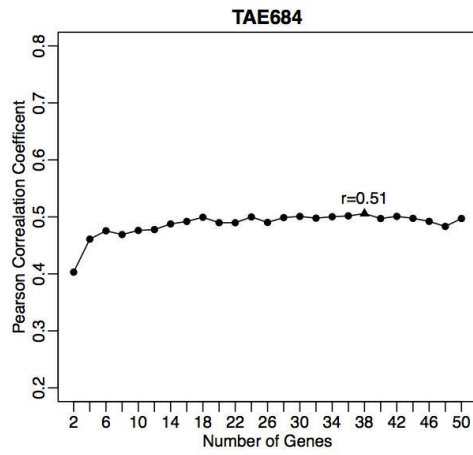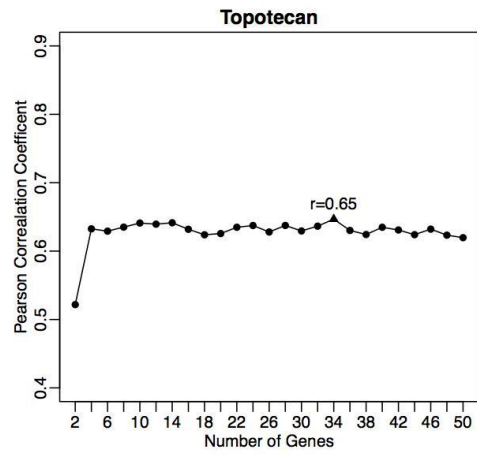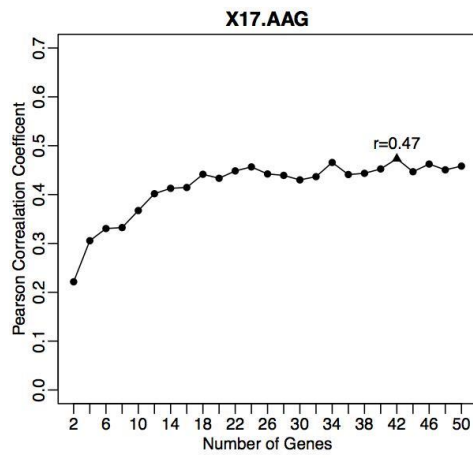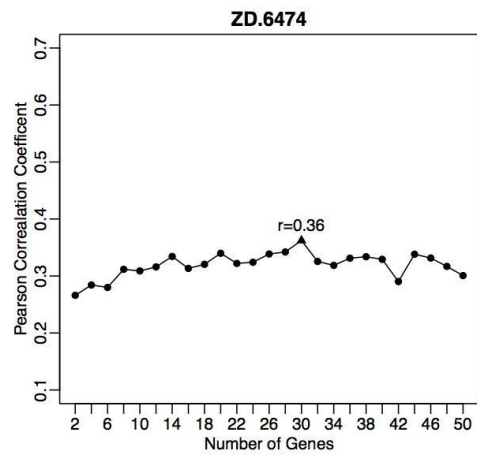

Supplement: Supplementary file 5 — Additional file 5: Fig. S1: Pearson correlation coefficients of predicted and true drug sensitivities at different numbers of recruited features for the other 20 drugs. [file 12911_2020_1240_MOESM5_ESM.pdf]
